# Cannabinoids synergize with carfilzomib, reducing multiple myeloma cells viability and migration

**DOI:** 10.18632/oncotarget.12721

**Published:** 2016-10-18

**Authors:** Massimo Nabissi, Maria Beatrice Morelli, Massimo Offidani, Consuelo Amantini, Silvia Gentili, Alessandra Soriani, Claudio Cardinali, Pietro Leoni, Giorgio Santoni

**Affiliations:** ^1^ School of Pharmacy, Experimental Medicine Section, University of Camerino, Camerino, Italy; ^2^ Department of Molecular Medicine, Sapienza University, Rome, Italy; ^3^ Clinica di Ematologia, Azienda Ospedaliero Universitaria Ospedali Riuniti di Ancona, Ancona, Italy; ^4^ School of Biosciences and Veterinary Medicine, University of Camerino, Camerino, Italy

**Keywords:** carfilzomib, THC, CBD, multiple myeloma, immuno-proteasome, combination therapy

## Abstract

Several studies showed a potential anti-tumor role for cannabinoids, by modulating cell signaling pathways involved in cancer cell proliferation, chemo-resistance and migration. Cannabidiol (CBD) was previously noted in multiple myeloma (MM), both alone and in synergy with the proteasome inhibitor bortezomib, to induce cell death. In other type of human cancers, the combination of CBD with Δ^9^-tetrahydrocannabinol (THC) was found to act synergistically with other chemotherapeutic drugs suggesting their use in combination therapy. In the current study, we evaluated the effects of THC alone and in combination with CBD in MM cell lines. We found that CBD and THC, mainly in combination, were able to reduce cell viability by inducing autophagic-dependent necrosis. Moreover, we showed that the CBD-THC combination was able to reduce MM cells migration by down-regulating expression of the chemokine receptor CXCR4 and of the CD147 plasma membrane glycoprotein. Furthermore, since the immuno-proteasome is considered a new target in MM and also since carfilzomib (CFZ) is a new promising immuno-proteasome inhibitor that creates irreversible adducts with the β5i subunit of immuno-proteasome, we evaluated the effect of CBD and THC in regulating the expression of the β5i subunit and their effect in combination with CFZ. Herein, we also found that the CBD and THC combination is able to reduce expression of the β5i subunit as well as to act in synergy with CFZ to increase MM cell death and inhibits cell migration. In summary, these results proved that this combination exerts strong anti-myeloma activities.

## INTRODUCTION

Increasing studies support the benefit of cannabinoids in cancer therapy, especially in terms of their effects in the induction of cell death, inhibition of proliferation and anti-metastatic activity noted in different human cancer *in vitro* and *in vivo* models [1, 2]. Cannabinoids are a family of compounds that exert their biological actions via a dependent-receptors mechanism, by binding mainly to Cannabinoid receptor type-1 and -2 (CB1, CB2) and Transient Potential Vanilloid type 1 and 2 (TRPV1, TRPV2) [3]. Moreover, receptors independent cannabinoids effects have also been described in cancer [1]. The most relevant effect of cannabinoids in cancers was investigated with Δ^9^-tetrahydrocannabinol (THC) and cannabidiol (CBD). THC and/or CBD were able to reduce cell proliferation and induce cell death in glioblastoma (GBM), lung and breast cancers, hepatocellular carcinoma and melanoma [4–10]. In addition, CBD has been shown to reduce viability, induce necrosis as well as synergize with bortezomib (BTZ) in reducing cell proliferation and cell survival pathways in multiple myeloma (MM) cell lines [11]. THC and CBD also show anti-inflammatory activities, by decreasing the release of pro-inflammatory cytokines (IFN-γ, IFN-β, IL-1 β, IL-6) and related transcription factors (such as NF-kB and STAT-3), in normal [12] and cancer cell lines, including MM [11]. Another important feature is that treatment with cannabinoids has been shown to reduce invasiveness of cancer cells as well as CXCR4-mediated migration of immune cells [13].

MM is a malignant disorder characterized by uncontrolled monoclonal plasma cell proliferation followed by the accumulation of malignant plasma cells in the bone marrow (BM), with possible escalation to anemia, osteolytic bone lesions, renal insufficiency, hypercalcemia and ultimately to extramedullary disease [14]. The prognosis of patients with MM has improved in the past decade, in respect of both progression-free survival (PFS) and overall survival (OS) [15], due to the introduction of a novel class of agents, such as immunomodulatory drugs (lenalidomide and pomalidomide) and proteasome inhibitors (BTZ and carfilzomib, CFZ) [16].

The constitutive proteasome (cPTS) and the immuno-proteasome (iPTS) are two major isoforms of proteasomes that have been described in humans. The cPTS, present in most cells, is composed by β5, β2 and β1 subunits [17]. The iPTS is comprised of related homologous protein subunits β1i, β2i, and β5i and it is predominantly expressed in cells of lymphoid origin. In these cells, exposure to interferon-γ (IFN-γ) or tumor necrosis factor-α (TNF-α) strongly and synergistically induces the expression of the β5i subunit [18]. During inflammatory states, the expression of these inducible ‘immunosubunits’ is strongly upregulated and the neosynthesis of cPTS is switched almost exclusively to the generation of the iPTS [18]. The cPTS has emerged as an important target in MM cancer therapy, leading to the approval of BTZ for newly diagnosed and relapsed/refractory MM [19, 20]. The reversible cPTS inhibitor BTZ, inhibits the cell cycle and induces apoptosis in MM cell lines, but is known to display hematologic toxicities (neutropenia and thrombocytopenia) and peripheral neuropathy [21]. So, to overcome these negative side effects and partially suppress BTZ resistance, a new generation of proteasome inhibitors was developed. CFZ increases safety and efficacy in MM treatment [22–24], and unlike BTZ, this drug creates irreversible adducts, specifically with the N-terminal threonine of the β5 and β5i subunits of cPTS and iPTS, respectively. CFZ also inhibits cell viability in different MM cell lines as well as patient-derived MM neoplastic cells by inducing apoptotic-signaling pathways [23]. Furthermore, CFZ shows enhanced anti-MM activity when compared with BTZ and it is also able to overcome resistance to BTZ in MM cells [23]. Acquired resistance to BTZ, in MM, can be the result of the acquisition of mutations in the β5 subunit and since the β5i counterpart does not harbor similar mutations, the down-regulation of iPTS in BTZ-resistant MM cell lines may provide a mechanism of escape [24, 25]. During end-stage of MM, malignant cells can survive and proliferate outside the microenvironment of the BM. The chemokine receptor CXCR4 and the CD147 receptor, which are up-regulated in MM plasmacells, have shown involvement in the recruitment of these cells to the BM [26].

Since at present there is no data concerning the potential effects of cannabinoids in the regulation of iPTS activity and migration in MM, herein, we evaluated the role of THC and CBD alone and in combination with CFZ, in regulating CFZ sensitivity, β5i expression and MM cell migration.

## RESULTS

### THC and THC-CBD combination induced cytotoxicity in MM cell lines

**Figure 1:**
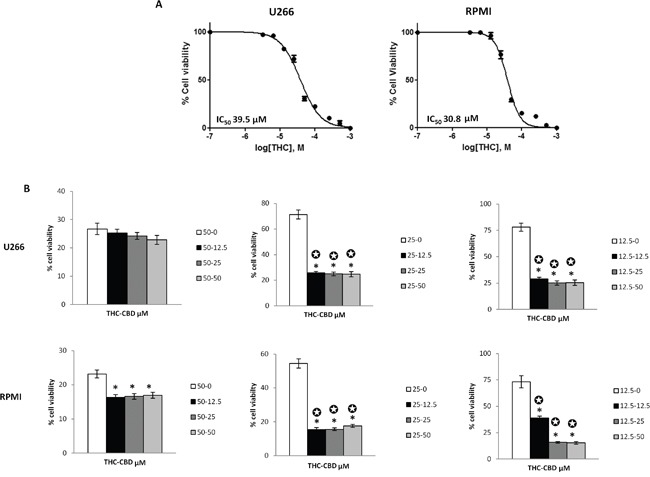
THC alone and in combination with CBD induces cytotoxicity in MM cell lines **A.** U266 and RPMI cell lines were treated with different doses of THC (from 0 to 1 mM). Cell viability was evaluated at 72 h post-treatments, by the MTT assay. Data shown are expressed as mean ± SE of three separate experiments. IC_50_ of THC in U266 and RPMI cell lines were indicated. **B.** THC and CBD act synergically in inducing cell cytotoxicity. U266 and RPMI cell lines were treated with different combinations of THC (12.5-50 μM) and CBD (0-50 μM). Cell viability was evaluated at 72 h post-treatments, by the MTT assay. Data shown are expressed as mean ± SD of three separate experiments. *p<0.05 vs THC alone treated cells. ✪ indicate synergism (C<1).

The effect of CBD in reducing cell viability was previously studied [11] in the U266 (IC_50_ = 19.8 μM) and in the RPMI (IC_50_= 22.4 μM) cell lines. In the present study, we treated U266 and RPMI cells with THC (up to 1 mM) for 72 h and percentage of cell viability was evaluated by the MTT assay. The results showed a dose dependent THC effect in both cell lines, with an IC_50_ of 39.5 μM and 30.8 μM in U266 and RPMI cells respectively (Figure 1A). Then, we determined the effects of different combinations of THC plus CBD to evaluate a potential synergism between the two cannabinoids, in both cell lines. The results showed that different doses of THC and CBD result in higher cytotoxicity when compared with THC alone (Figure 1B) [11], and that THC (25 μM and 12.5 μM) acts synergically (CI<1) with CBD (50, 25 and 12.5 μM), inducing higher cytotoxic effects compared with single doses (Figure 1B). So, we decided to work with the lowest doses of 12.5 μM for CBD and 12.5 μM for THC in the following experiments.

**Figure 2:**
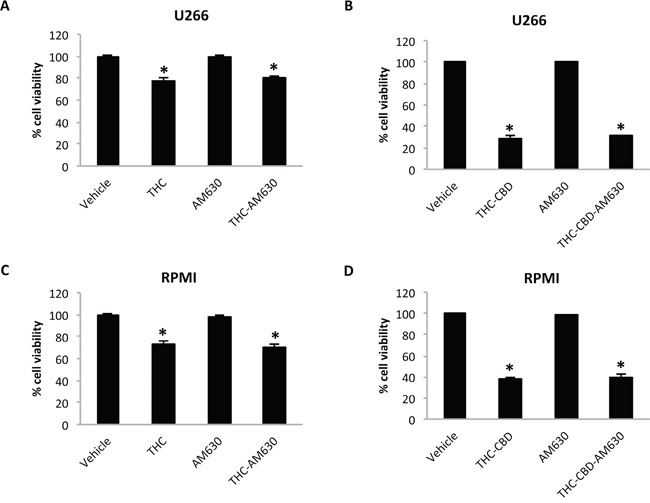
THC and THC-CBD cytotoxic effects are CB2 receptor independent U266 and RPMI cells were treated with AM630 (20 μM) alone or in combination with THC 12.5 μM **A, C**, or with 12.5 μM CBD plus 12.5 μM THC **B, D.** Cell viability was evaluated by using the MTT assay. Data shown are expressed as mean ± SD of three separate experiments. *p<0.05 vs vehicle treated cells.

In addition, the cytotoxic effect of CBD and THC alone and in combination was demonstrated not to be CB2 receptors dependent, as evidenced by pre-treating MM cell lines with 20 μM AM630 (CB2 antagonist) followed by THC alone or in combination with CBD (Figure 2).

### THC-CBD combination induces cell cycle arrest in MM cell lines

**Figure 3:**
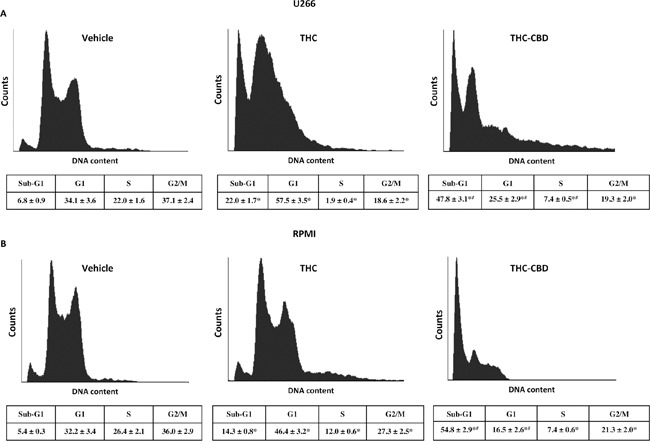
THC alone and THC-CBD combination increase the sub-G1 phase in U266 and RPMI cell lines **A, B.** Cell cycle analysis of U266 and RPMI cell lines treated with THC (12.5 μM) alone or in combination with CBD (12.5 μM). Cell cycle was performed using the PI incorporation assay and FACS analysis, after 48 h post-treatments. Histograms are representative of one of three separate experiments. The values represent the percentage of cells in each phase and are expressed as mean ± SD. *p<0.05 vs vehicle treated cells; #p<0.05 vs THC treated cells.

The effect of CBD in blocking cell cycle in G1 phase (38% in U266, 42% in RPMI) was previously proved [11]. So we evaluated the role of THC alone or in combination with CBD in influencing the cell cycle, in both MM cell lines. The cell cycle phases were analysed by propidium iodide (PI) staining and FACS analysis in both cell lines. The results showed that THC was able to induce cell accumulation in the G1 phase, starting from 24 h post-treatment, accompanied by accumulation in the sub-G1 phase (hypodiploid DNA) at 48 h post-treatment, compared with their respective control (Supplementary Figure 1; Figure 3). The THC-CBD combination was statistically more effective in increasing the G1 cell population and the sub-G1 phase at 24 h post-treatment and in augmenting cell accumulation in the sub-G1 phase at 48 h, compared with THC and CBD [11] when used alone (Figure 3, Supplementary Figure 1). This data suggested that the THC-CBD combination was more effective than THC and CBD used as single agents in inducing cell death, in both cell lines.

### THC-CBD combination induces autophagic-cell death in MM cell lines

**Figure 4:**
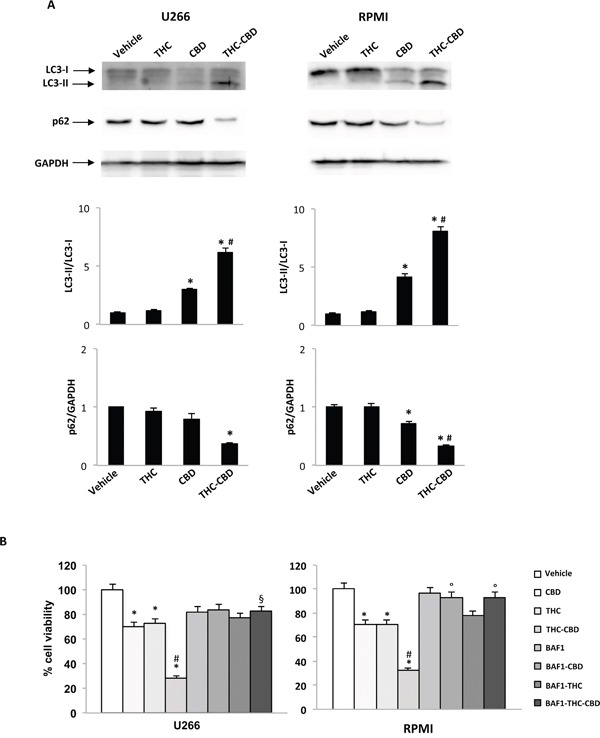
THC-CBD combination induces autophagic-cell death in MM cell lines **A.** U266 and RPMI were treated with CBD (12.5 μM), THC (12.5 μM) alone and in combination. Lysates of treated cells were separated on SDS-PAGE and probed with anti-LC3, anti-p62 and anti-GAPDH Abs. Blots are representative of one of three separate experiments. Bars represent the densitometric analysis. *p<0.01 vs vehicle and THC treated cells; #p<0.01 vs CBD treated cells. **B.** U266 and RPMI cell lines were pretreated with BAF1 (50 nM) for 1 h and then treated with CBD (12.5 μM), THC (12.5 μM) alone and in combination for 72 h. Data shown are expressed as mean ± SD of three separate experiments. *p<0.05 vs vehicle treated cells; ^#^p<0.05 vs THC, CBD and BAF1, alone or incombination; ^§^p<0.05 vs THC-CBD; °p<0.05 vs THC, CBD and THC-CBD.

This study investigated whether increasing of the sub-G1 cell accumulation by THC-CBD treatment was due to an autophagic-cell death process. We examined the conversion of the soluble form of LC3 (LC3-I) to the lipidated and autophagosome-associated form (LC3-II), marker of autophagy activation, in THC, CBD and THC-CBD treated cells after 24 h of treatment, using western blot analysis. We found that CBD alone induces a slight increase of LC3-II/LC3-I ratio, THC has no effect, while the THC-CBD combination strongly augments the levels of the cleaved LC3-II form and the LC3-II/LC3-I ratio, compared with single treatments (Figure 4A). We also evaluated the variation of p62 levels. The results evidenced that THC-CBD combination is able to strongly reduce the p62 protein levels, with respect to THC and CBD alone in MM-treated cells (Figure 4A). Additionally, to determine the role of the autophagic pathway in THC and CBD effects we pre-treated the cells with the autophagic inhibitor bafilomycin A1 (BAF1). By MTT assay we found that CBD and THC-CBD cytotoxic effects were reversed by the pre-treatment with BAF1 (Figure 4B).

**Figure 5:**
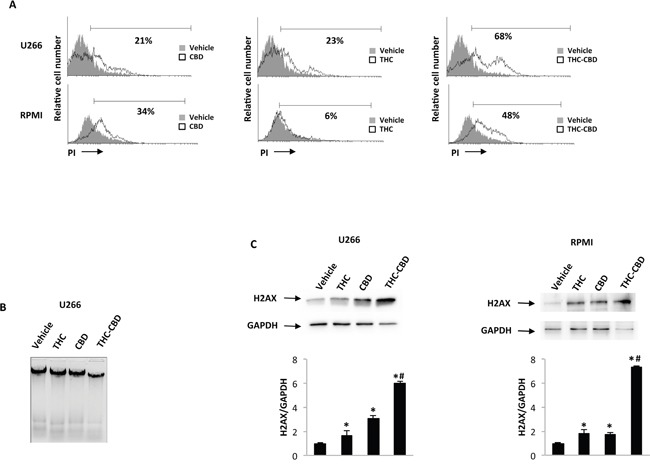
CBD-THC combination induces necrosis U266 and RPMI cell lines were treated for 72 h with CBD (12.5 μM), THC (12.5 μM) alone and in combination. **A.** The percentage of PI positive cells were determined by FACS analysis. Histograms are representative of one of three separate experiments. Data are expressed as percentage of PI positive cells with respect to vehicle treated cells. **B.** Representative agarose gel electrophoresis of DNA extracts obtained from U266 treated cells for the assessment of DNA fragmentation. **C.** H2AX protein levels were determined by western blot analysis. H2AX densitometry values were normalized to GAPDH used as loading control. Blots are representative of one of three separate experiments. Data shown are expressed as mean ± SD of three separate experiments. *p<0.05 vs vehicle treated cells; #p<0.01 vs THC or CBD treated cells.

Using PI staining and FACS analysis we also evidenced that THC-CBD combination induces higher necrotic cell death compared with THC and CBD alone, at 48 h post-treatments, in both MM cell lines (Figure 5A). Furthermore, we evidenced augmented levels of damaged DNA after addition of THC-CBD combination with respect to the single treatment as demonstrated by genomic DNA fragmentation analysis (Figure 5B). We also investigated the presence of γ-H2AX (H2AX), a phosphorylated variant of histone 2A that is associated with DNA double-strand breaks. Immunoblots showed that THC and CBD in both cell lines are able to induce increased levels of the phosphorylated form of H2AX (Figure 5C) at 24 h post-treatments, and THC-CBD further improves the H2AX levels respect to the single treatments, in both MM cell lines (Figure 5C).

### Effect of THC-CBD in regulating the β5i subunit in MM cell lines

**Figure 6:**
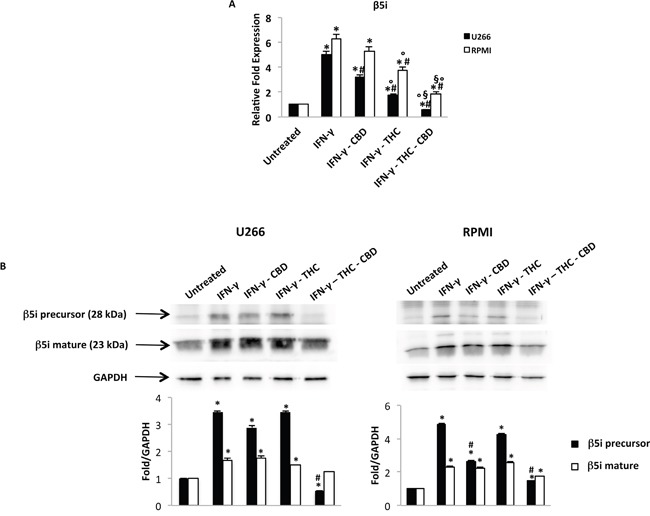
Regulation of the β5i subunit by THC and CBD in MM cell lines stimulated with IFN-γ U266 and RPMI cells were treated with IFN-γ 100 U/ml for 24 h. Then cells were treated with THC (12.5 μM), CBD (12.5 μM) alone and in combination for additional 24 h. **A.** The β5i mRNA levels were determined by qRT-PCR. GAPDH was used for normalization. Data are expressed as relative fold with respect to vehicle treated cells used as the control. Data are expressed as mean ± SD. *p<0.01 vs untreated; ^#^p<0.01 vs IFN-γ; °p<0.01 vs IFN-γ-CBD; ^§^p<0.05 vs IFN-γ-THC. **B.** The levels of the precursor and mature form of the β5i subunit were analysed by western blot. GAPDH was used as the loading control. Blots are representative of three separate experiments. Bars represent the densitometric analysis. *p<0.05 vs untreated cells; #p<0.05 vs IFN-γ treated cells.

We evaluated a potential role of THC-CBD in regulating the β5i subunit. So, U266 and RPMI cell lines were treated with CBD and THC, after 24 h exposure to IFN-γ (100 U/ml). Using qRT-PCR, we showed that the THC-CBD combination strongly reduces the β5i increased expression level induced by IFN-γ, while low effects were observed with single CBD and THC treatments respect to IFN-γ alone (Figure 6A). At protein levels, the expression of the precursor and mature form of β5i was examined by western blot analysis. Results evidenced that the administration of IFN-γ increases both the precursor and the mature form of β5i in MM-treated compared with MM non-treated cells. Moreover, THC and CBD alone had low efficacy in reducing β5i, while the THC-CBD combination impaired the expression of both forms, in U266 and RPMI cell lines (Figure 6B).

### THC-CBD combination synergizes with CFZ in reducing cell viability in MM cell lines

**Figure 7:**
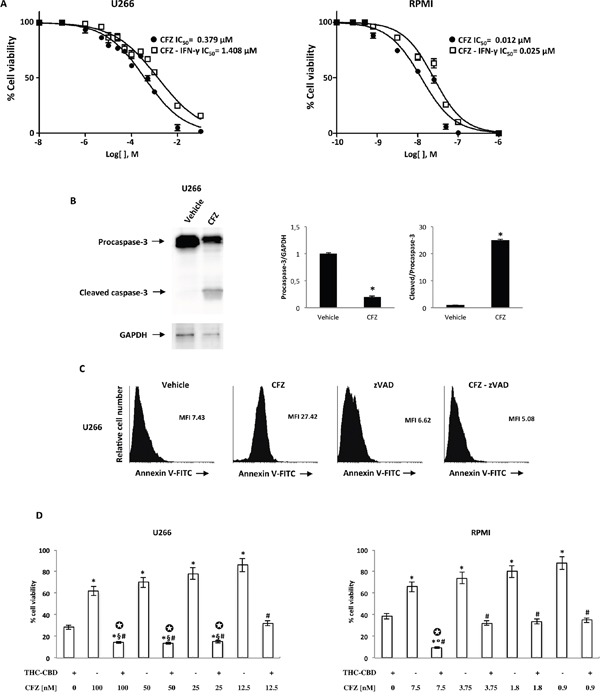
The effect of CFZ alone or in combination with THC-CBD on cell viability **A.** U266 and RPMI cell lines were cultured for 72 h with different doses of CFZ in the presence or absence of IFN-γ (100 U/ml). Cell viability was determined by the MTT assay. Data shown are expressed as mean ± SE of three separate experiments. **B.** Lysates from the U266 cell line treated with CFZ 100 nM for 72 h, were analyzed for caspase-3 protein level by western blot analysis. GAPDH protein levels were evaluated as the loading control. Blots are representative of three separate experiments. Bars represent the densitometric analysis. *p<0.01 vs vehicle treated cells. **C.** U266 cell lines were pre-treated with 5 mM zVAD for one h and then treated with 100 nM CFZ for 72 h. The percentage of Annexin V positive cells were determined by FACS analysis. Histograms are representative of one of three separate experiments. MFI, mean fluorescence intensity. **D.** U266 and RPMI cell lines were treated with THC-CBD in combination with different doses of CFZ. Cell viability was evaluated by the MTT assay. Data shown are expressed as mean ± SD of three separate experiments. *p<0.01 vs THC-CBD treated cells; ^#^p<0.01 vs CFZ alone; ^§^p<0.01 vs THC-CBD-CFZ vs THC-CBD-CFZ (12.5 nM); ^°^p<0.01 vs THC-CBD-CFZ (7.5 nM) vs THC-CBD-CFZ (3.75, 1.8, 0.9 nM). ✪ indicates synergism (C<1).

To evaluate the potential inhibitory effect of immuno-proteasome inhibitor CFZ on cell viability, U266 and RPMI cell lines were exposed to increasing concentration of CFZ in presence or absence of IFN-γ, and cell viability was evaluated by the MTT assay 72 h post-treatment. As shown (Figure 7A), CFZ was able to reduce cell viability in both MM cell lines, with IC_50_ 0.379 μM and 0.012 μM in U266 and RPMI, respectively. Moreover, stimulation with IFN-γ reduced CFZ sensitivity in both cell lines (U266 IC_50_= 1.426 μM; RPMI IC_50_= 0.026 μM). To understand the mechanism underlying the effect of CFZ on MM cell viability, we evaluated whether CFZ was able to influence cell cycle progression in MM cell lines. Using PI staining, cell cycle phases were determined in CFZ-treated cells after 24 h of treatment, by FACS analysis. The results showed that CFZ induced a rapid accumulation in sub-G1 phase in MM cell lines (Supplementary Figure 2). These results demonstrated that CFZ was able to induce cell death with minimal effect on cell cycle, in both cell lines. Then, we investigated on the role of caspase-3 in CFZ-induced apoptosis in U266 and RPMI cells. Both cell lines were treated with CFZ for 72 h and western blot analysis was performed to evaluate caspase-3 activation. As shown, CFZ was able to increase cleaved caspase-3 levels in MM cell lines (Figure 7B, Supplementary Figure 3A). Moreover, the role of caspase-3 in CFZ-induced apoptosis was further confirmed by pre-treating U266 and RPMI cell lines with the caspase-3 inhibitor z-VAD (5 mM) for 1 h prior to treat cells with CFZ for an additional 72 h. FACS analysis demonstrated that CFZ increased Annexin V^+^ cells, while z-VAD reduced CFZ-induced apoptosis in both cell lines (Figure 7C, Supplementary Figure 3B). In conclusion, these results revealed a pro-apoptotic effect of CFZ in U266 and RPMI cell lines. Both CFZ alone and THC-CBD combination reduce cell viability; therefore, we evaluated the effect of CFZ plus THC-CBD combination on MM cell viability. RPMI and U266 cells were treated with different doses of CFZ (0.9 up to 7.5 nM doses for RPMI, 12.5 up to 100 nM doses for U266) in combination with THC-CBD. The results showed that most of the combinations strongly reduce cell viability compared with single treatments in both cell lines (Figure 7D). Furthermore, we evidenced that THC-CBD combination acts synergically (CI<1) with CFZ (50, 25 and 12.5 nM in U266; 7.5 nM in RPMI) to induce cytotoxic effects.

### THC-CBD in combination with CFZ inhibits cell migration in MM cell lines

**Figure 8:**
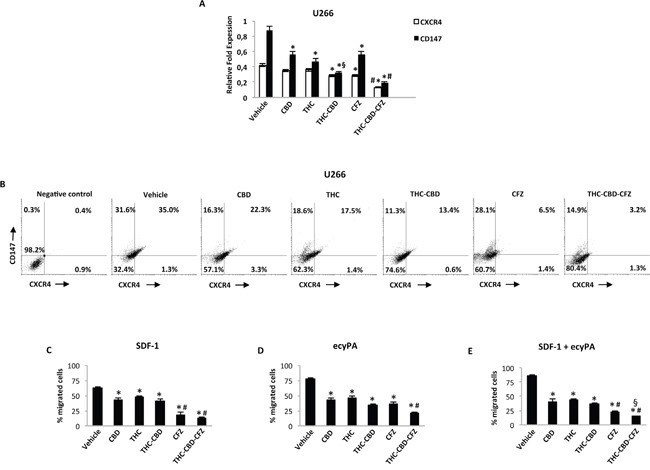
THC, CBD and CFZ inhibit cell migration in U266 cell line **A.** U266 cells were treated with CBD 12.5 μM, THC 12.5 μM, CFZ 100 nM alone or in combination for 24 h. CXCR4 and CD147 mRNA levels were determined by qRT-PCR. GAPDH was used for normalization. Data are expressed as relative fold with respect to vehicle treated cells used as control. Data are expressed as mean ± SD. *p<0.01 vs vehicle; ^#^p<0.01 vs THC, CBD, CFZ alone and CBD-THC;^§^p<0.05 vs CBD, THC. **B.** CXCR4 and CD147 expression was analyzed by fow cytometry on U266 cell line treated as described above. Representative dot plots illustrate the double fluorescence. Numbers represent the percentage of cells in each quadrant. Data are representative of 1 of 4 independent experiments. **C-E.** Cell migration was analysed by transwell migration assays. Data represent the percentage of migrated U266 cells and are expressed as mean ± SD. In C: *p<0.01 vs vehicle; ^#^p<0.01 vs THC, CBD, CBD-THC. In D: *p<0.01 vs vehicle; ^#^p<0.01 vs THC, CBD, THC-CBD, CFZ. In E: *p<0.01 vs vehicle; ^#^p<0.01 vs THC, CBD, THC-CBD; ^§^p<0.01 vs CFZ.

We first evaluated the expression of CXCR4 and CD147 in U266 and RPMI cell lines by qRT-PCR and FACS analysis. The qRT-PCR results showed that CXCR4 is expressed, although at lower levels in relation with CD147 levels, in both RPMI and U266 cell lines (Supplementary Figure 4A, Figure 8A). FACS analysis confirmed the qRT-PCR data, since 95% and 42% of RPMI and 66.6% and 36% of U266 cell population express CD147 and CXCR4. All CXCR4^+^ RPMI and U266 cells were CD147^+^ (Supplementary Figure 4B, Figure 8B). Then, we evaluated the effect of CBD, THC and CFZ alone or in combination, in regulating CXCR4 and CD147 expression, in both MM cell lines. Cells were treated with a single dose of compounds alone or in combination for 24 h, and mRNA transcripts and protein levels were analyzed by qRT-PCR and FACS analysis. qRT-PCR showed that CXCR4 and CD147 transcript levels decrease, and that the combination of CBD-THC plus CFZ was most effective in reducing CXCR4 and CD147 mRNA expression in both MM cell lines (Supplementary Figure 4A, Figure 8A). The qRT-PCR results were then confirmed by FACS analysis. A substantial decrease of both CXCR4^+^ and CD147^+^ and CXCR4^+^CD147^+^ cells, compared with the respective control cells was observed in U266 and RPMI MM cell lines (Figure 8B, Supplementary Figure 4B), with the THC-CBD plus CFZ being more effective in reducing the percentage of CXCR4^+^CD147^+^ and CD147^+^ cell phenotype. To further investigate the consequence of this effect, we treated for 24 h U266 and RPMI cells with the appropriate dose of CBD, THC and CFZ alone and in combination and then measured cell migration. The results showed that CBD, THC and CFZ both alone and in combination, reduce the SDF-1-, eCyPA- and SDF-1/eCyPA-mediated chemotaxis, compared with vehicle-treated cells (Figure 8C, Supplementary Figure 4C). In conclusion, these results suggest that CBD, THC and CFZ alone and in combination reduced both the expression of CXCR4 and CD147 as well as their chemotactic activity induced by SDF-1-, and eCyPA in MM cell lines.

## DISCUSSION

Over the last twenty years the antitumor benefits afforded by cannabinoids have been proved in different human cancer cell lines and *in vivo* preclinical models [1]. The main effects of cannabinoids in impairing tumor progression were related to their anti-proliferative, pro-cell death and anti-migratory activities, which were noted in solid and haematological cancers. In GBM both CBD and the THC-CBD combination, were found to reduce cell viability and induce apoptosis *in vitro* and in GBM xenografts [4, 27]. CBD induces apoptotic cell death *in vitro* in A549, H460 lung cancer cell lines and in primary cells from patients with lung cancer and causes tumor regression in A549-xenografted nude mice [28]. In breast cancer, THC inhibits cell proliferation by blocking the cell cycle and inducing apoptotic cell death [29, 30], while CBD inhibits AKT and mTOR signaling inducing autophagic-cell death [31].

In multiple myeloma, our previous findings demonstrated that CBD reduced cell proliferation and induced necrotic cell death [11]. In the present study, our data on THC and mainly on the THC-CBD combination as stimulatory factors of autophagic-dependent cell death in MM cell lines, support previous data regarding the efficacy of cannabinoids as anti-tumoral drugs, in different human cancer models.

For cannabinoids, different experimental data suggested that the combined administration of cannabinoids with other anti-cancer drugs, could act synergistically, to reduce tumor growth and chemoresistance. In GBM, temozolomide and carmustine, exert anti-tumor activity and the combination with CBD or THC-CBD synergistically increases GBM cells death both *in vitro* and *in vivo*, overpowering resistance mechanisms and lowering the chemotherapeutic doses, thereby leading to few adverse events [4, 32]. In another study it was reported that the combination of THC with cytotoxic agents (cytarabine, doxorubicin, vincristine) increased apoptosis in leukemia cells [33] and CBD was shown to enhance the ability of triple negative breast cancer subtype cells to uptake doxorubicin and significantly enhance its anti-tumorigenic efficacy [34]. In MM cells, the CBD and BTZ combination was found to be more effective compared with BTZ alone and to act synergistically in inducing cell death [11]. Herein we investigated the effect of CBD and THC in combination with CFZ, showing a synergistic effect between the three drugs, supporting the fact that combining THC-CBD with established cytotoxic agents should result in a higher level of anticancer activity compared with that of cytotoxic agents acting alone.

CFZ was demonstrated to induce apoptosis in the ANBL-6 cell line, increasing the caspase-3 activity confirmed by the effect of zVAD that blocked CFZ-stimulated apoptosis [23]. Herein, we confirmed the caspase-3 role in CFZ-induced apoptosis, suggesting the caspase-3 driven apoptosis is a common mechanism of action of CFZ in MM cell lines. A mechanism of CFZ resistance determined in MM cells was related to the expression levels of β5i. Moreover, the role of IFN-γ in exchanging the cPTS subunits for iPTS subunits was first demonstrated in J111 leukemia cells [35]. Our findings demonstrated that, in IFN-γ-treated MM cell lines the levels of the β5i subunit increased and this treatment augmented the CFZ resistance. THC-CBD treatments, by reducing β5i subunit both at transcriptional and translational levels, induced inhibition of the CFZ target β5i subunit, indicating cannabinoids as potential drugs for overcoming CFZ resistance mechanisms.

Another potential anti-cancer activity of cannabinoids is related to their capacity to reduce cell migration, as observed in glioma [36], breast [7] and lung cancer [8], while no data was reported regarding MM. The homing of MM cells in bone marrow is associated with the progression of MM and patient's survival [37]. Factors implicated in bone marrow homing of MM cells include the chemokine receptor CXCR4 and CD147 and their ligands SDF-1 and eCyPA [27, 38]. Clinically, expression of CXCR4 protein in tumors is used to predict cancer aggressiveness, survival probability and metastasis-associated mortality [39, 40]. Few data about cannabinoids and CXCR4, indicate that CB2 modulates the CXCR4-induced transendothelial migration of T cells, altering multiple immune and inflammatory responses [41], and CB2 agonist specifically reduced CXCR4-mediated migration [13]. Regarding CD147, it has critical roles in intercellular communication involved in chronic inflammation, tumor metastasis and angiogenesis [42–44]. Recently, CD147 has been correlated with the progression of various carcinomas and haematological cancers, as MM [45, 46]. In MM, the CD147 expression increases with disease progression, and eCyPB induced the proliferation and homing of MM cells [45]. Therefore, developing agents that can inhibit the action of CXCR4 or CD147, in early and advanced stages of cancer may be effective in preventing and managing metastasis [47]. In this study, we showed that CD147 was the main represented receptor respect to CXCR4 and that both cannabinoids and CFZ alone and in combination were able to reduce CD147 and CXCR4 expression levels. To further confirm the role of these drugs in decreasing MM cell migration, we applied a migration assay, which further confirm that, mainly the triple combination was able to reduce this phenomena, in both cell lines. While in MM the role of CFZ and cannabinoids in inhibiting migration has never been evaluated, recently the anti-migration activity of CFZ was evidenced in GBM cell lines [48], suggesting the CFZ could share a new potential application as anti-metastatic drugs, and probably with major effect when combined with cannabinoids, at least in MM. In conclusion, this study adds further support to the hypothesis that cannabinoids can have a role in the cancer management. To note, the effective doses of cannabinoids and CFZ used in this *in vitro* study are coherent with dosages used in clinical setting, as reported in clinical trials with THC/CBD for combination with anti-tumoral therapy [49], and for CFZ in MM patients [51], as example. In both clinical cases, the *in vivo* doses of THC/CBD and CFZ used in human trials, were obtained converting their *in vitro* cytotoxic concentrations that were similar to our effective doses.

Therefore, a combination therapy including cannabinoids and chemotherapeutic drugs could allow the reduction of chemotherapeutical doses administered in patients, without affecting the antitumoral therapy.

## MATERIALS AND METHODS

### Cell culture

U266 and RPMI8226 (RPMI) MM cell lines were purchased from ATCC (LGC Standards, Milan, IT). Cell authentication was performed by IST (Genova, Italy). Cell lines were cultured in RPMI1640 medium (Lonza, Milan, IT) supplemented with 10% foetal bovine serum (FBS), 2 mM L- glutamine, 100 IU/ml penicillin, 100 μg/ml streptomycin and 1 mM sodium pyruvate. Cell lines were maintained at 37°C with 5% CO_2_ and 95% humidity.

### Compounds

Pure CBD and THC were supplied from GW Pharmaceuticals (batch CBD/160810; batch THC/CG/1301). CBD and THC were dissolved in ethanol. AM630 and bafilomycin A1 (Tocris Bioscience, Bristol, UK) were dissolved in DMSO. z-VAD, CFZ and IFN-γ (Sigma Aldrich, Sant Luis, MO, USA) were dissolved in distilled water.

### MTT assay

U266 and RPMI cell lines (4 × 10^4^ cells/ml) were seeded in 96-well plates, in a final volume of 100 μl/well. After one day of incubation, compounds or vehicles were added. At least four replicates were used for each treatment. At the indicated time point, cell viability was assessed by adding 0.8 mg/ml of 3-[4,5-dimethylthiazol-2-yl]-2,5 diphenyl tetrazolium bromide (MTT) (Sigma Aldrich) to the media. After 3 h, the plates were centrifuged, the supernatant was removed, and the pellet was solubilized with 100 μl/well DMSO. The absorbance of the samples against a background control (medium alone) was measured at 570 nm using an ELISA reader microliter plate (BioTek Instruments, Winooski, VT, USA). Synergistic activity of the THC-CBD combinations was determined by the isobologram and combination index (CI) methods (CompuSyn Software, ComboSyn, Inc. Paramus, NJ 2007). The CI was used to express synergism (CI < 1), additivity (CI = 1) or antagonism (CI > 1) and was calculated according to the standard isobologram equation [49].

### Cell cycle analysis

U266 and RPMI cell lines (4 x10^4^ cells/ml) were incubated with the appropriate drugs for up to 72 h. Cells were fixed for 1 h by adding ice-cold 70% ethanol and then washed with staining buffer (PBS, 2% FBS and 0.01% NaN_3_). The cells were treated with 100 μg/ml ribonuclease A solution (Sigma Aldrich), incubated for 30 min at 37°C, stained for 30 min at room temperature with propidium iodide (PI) 20 μg/ml (Sigma Aldrich) and analysed on a FACScan flow cytometer using CellQuest software.

### Apoptosis assay

The exposed phosphatidylserine on the U266 and RPMI cells membrane surface was detected by Annexin V staining and cytofluorimetric analysis. Briefly, 4 × 10^4^ cells/ml were treated with different doses of the appropriate drugs for a maximum of 72 h. Four replicates were used for each treatment. After treatment, the cells were stained with 5 μl of Annexin V FITC (Vinci Biochem, Vinci, Italy) for 10 min at room temperature, washed once with binding buffer (10 mM N- (2- Hydroxyethyl) piperazine-N0-2-ethanesulfonic acid [HEPES]/sodium hydroxide, pH 7.4, 140 mM NaCl, 2.5 mM CaCl2) and analysed on a FACScan flow cytometer using CellQuest software.

### PI staining

After treatment with the appropriate drugs for a maximum of 72 h, 4 × 10^4^ U266 and RPMI cells/ml, were incubated in a binding buffer containing 20 μg/ml PI for 10 min at room temperature. The cells were then analysed by flow cytometry using CellQuest software.

### Western blot analysis

U266 and RPMI cell lines were lysed in a buffer containing a protease inhibitor cocktail (Sigma Aldrich). Lysates were resolved by sodium dodecyl sulphate polyacrylamide gel (8-14%) and transferred onto Hybond-C extra membranes (GE Healthcare, Munich, Germany). Non-specific binding sites were blocked with 5% low-fat dry milk in phosphate-buffered saline 0.1% Tween 20 for l h. Blots were incubated with the primary Abs: anti-iβ5 subunit (1:1000, Cell Signaling, Denver, CO, USA), rabbit anti-LC3 (2 μg/ml, Novus Biologicals, Littleton, CO, USA), rabbit anti-caspase-3 (1:1000, Cell Signaling), rabbit anti-p62 (1:1000, Cell Signaling), rabbit anti-H2AX (1:1000, Cell Signaling) and mouse anti-glyceraldehydes-3-phosphate dehydrogenase (GAPDH, 1:3000, OriGene, Rockville, MD, USA) Abs overnight and then incubated with their respective HRP-conjugated anti-mouse and anti-rabbit (1:2000, Cell Signaling) Abs for 1 h. The detection was performed using the LiteAblot PLUS or the LiteAblot TURBO (EuroClone, Milano, Italy) kits, and densitometric analysis was carried out by a Chemidoc using the Quantity One software (Bio-Rad, Hercules, CA, USA).

### DNA fragmentation assay

Electrophoresis of DNA was performed to assess DNA fragmentation as an indicator of necrosis and apoptosis. Briefly, 4 × 10^4^ cells/ml were treated with the appropriate compounds for 72 h, and the genomic DNA was extracted using a DNA extraction kit (Qiagen, Hilden, Germany). The purified samples were then subjected to electrophoresis on 1.25% agarose gel, stained with ethidium bromide. Ultraviolet spectroscopy at 302 nm was used to obtain the results.

### RT-PCR analysis

Total RNA was extracted with the RNeasy Mini Kit (Qiagen), and cDNA was synthesized using the High-Capacity cDNA Archive Kit (Applied Biosystems, Foster City, PA) according to the manufacturer's instructions. Quantitative real-time polymerase chain reactions (qRT-PCR) for iβ5, CXCR4 and CD147 were performed using the iQ5 Multicolor Real-Time PCR Detection System (Bio-Rad, Hercules, CA). PCR reaction was performed with RT^2^SYBRGreen qPCT mastermix (Qiagen) using 1 μl of cDNA for reaction, following the amplification protocol described in the manufacture's instruction. RT^2^ qPCR Primer assays (Qiagen) were used for target gene amplification. All samples were assayed in triplicates in the same plate. Measurement of GAPDH levels was used to normalize mRNA contents, and target gene levels were calculated by the 2^−ΔΔCt^ method.

### Cell migration assay

U266 and RPMI cell lines were treated with the appropriate drugs for 72 h and cell migration was evaluated by the 96 wells cell migration assay (Trevigian, MD, USA) according to the manufacturer's instructions. SDF-1, eCyPA and SDF-1-eCyPA in combination were added to the bottom chamber as chemotaxis inducing agents. Data from the standard curve were used to determine the number of cells that had migrated, as well as the percentage cell migration.

### Statistical analysis

The statistical significance was determined by analysis of variance (ANOVA) or Student's t test. The statistical analysis of IC_50_ levels was performed using Prism 5.01 (Graph Pad). Data from untreated cells were omitted because no differences were observed between vehicle-treated and untreated cells.

## SUPPLEMENTARY MATERIALS FIGURES


