# Supplementary material for: Cannabinoids synergize with carfilzomib, reducing multiple myeloma cells viability and migration
Source: Oncotarget. 2016 Oct 18;7(47):77543–57. doi: 10.18632/oncotarget.12721 (PMC5363603; doi:10.18632/oncotarget.12721)
Supplement: Supplementary file 1 [file oncotarget-07-77543-s001.pdf]

Cannabinoids synergize with carfilzomib, reducing multiple myeloma cells viability and migration

SUPPLEMENTARY FIGURES

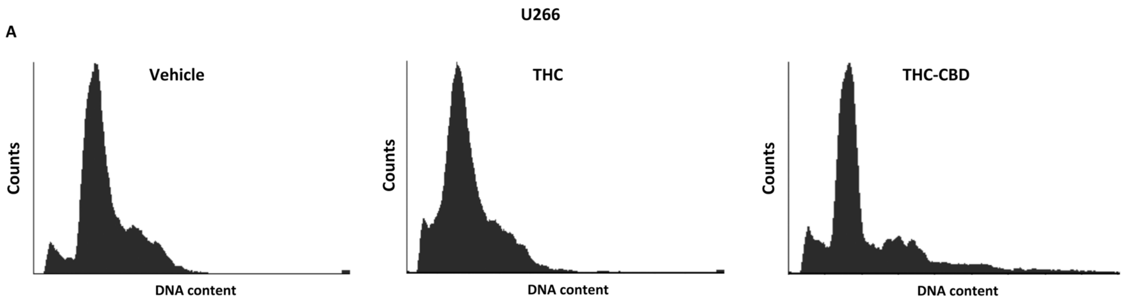

Vehicle

Sub-G1	G1	S	G2/M
5.4 ± 0.4	32.6 ± 3.6	23.2 ± 1.6	38.8± 2.4

THC

Sub-G1	G1	S	G2/M
7.8 ± 0.3	62.7 ± 3.0*	1.2 ± 1.4*	28.3 ± 2.2

THC-CBD

Sub-G1	G1	S	G2/M
21.4 ± 2.0*#	52.5 ± 2.9*#	6.5 ± 0.5*	19.6 ± 2.1*#

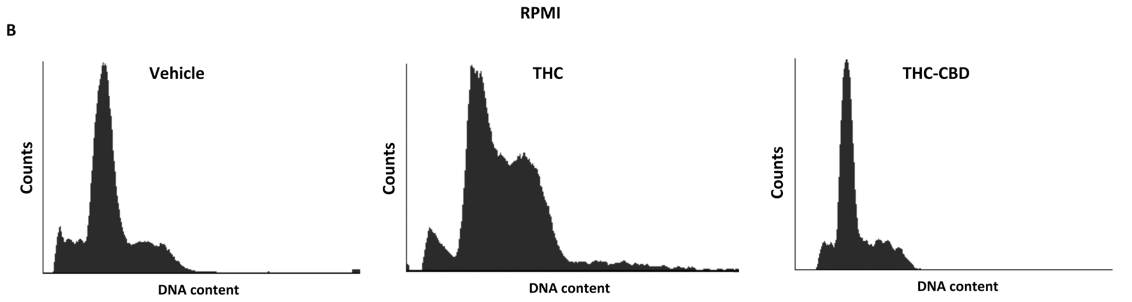

Vehicle

Sub-G1	G1	S	G2/M
6.4 ± 0.3	36.3 ± 3.0	20.8 ± 2.3	36.5 ± 2.9

THC

Sub-G1	G1	S	G2/M
13.3± 0.8	42.4 ± 2.6	14.0 ± 0.6	30.3± 2.1

THC-CBD

Sub-G1	G1	S	G2/M
12.8 ± 2.9*	57.5 ± 2.6#	7.4 ± 0.6*#	22.3 ± 2.0*

Supplementary Figure S1: THC alone and THC-CBD combination influence cell cycle in U266 and RPMI cell lines. Cell cycle analysis of U266 and RPMI cell lines treated with THC (12.5 µM) alone or in combination with CBD (12.5 µM). Cell cycle was performed by the PI incorporation assay and FACS analysis, after 24 h post-treatments. Histograms are representative of one of three separate experiments. The values represent the percentage of cells in each phase and are expressed as mean ± SD. *p<0.05 vs vehicle treated cells; #p<0.05 vs THC treated cells.

U266

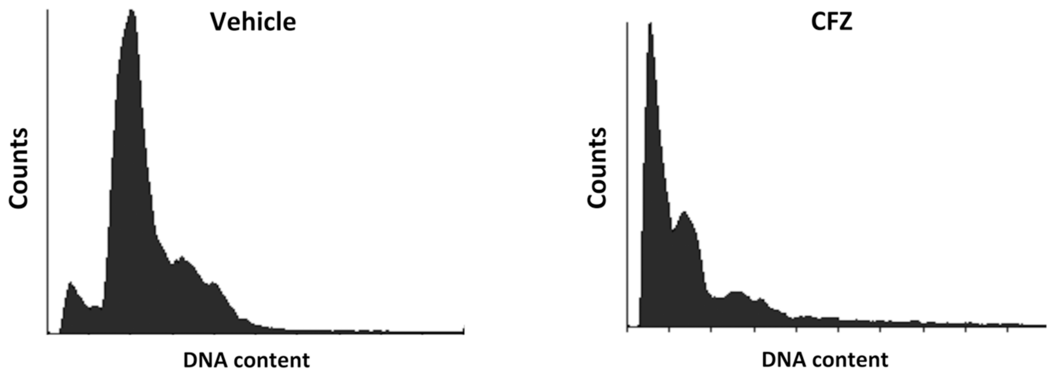

Sub-G1	G1	S	G2/M
6.4 ± 0.4	32.5 ± 3.0	20.0 ± 1.8	41.1 ± 3.4

Sub-G1	G1	S	G2/M
56.8 ± 4.1*	22.1 ± 2.6*	9.0 ± 0.6*	12.1 ± 1.4*

RPMI

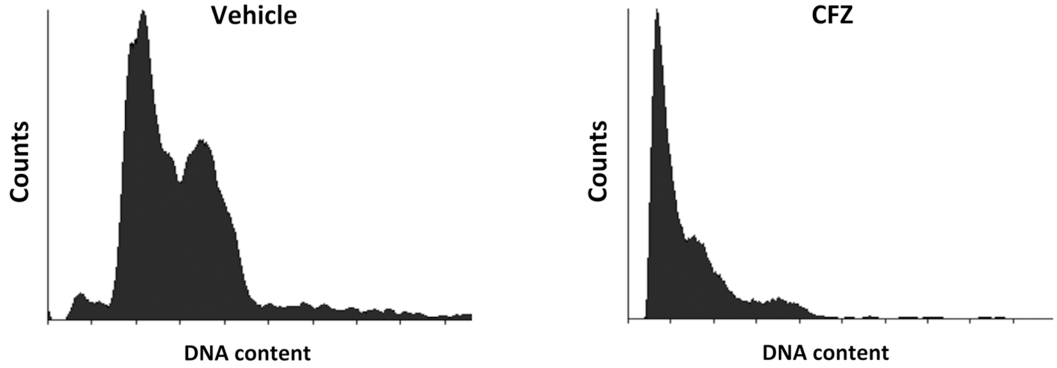

Sub-G1	G1	S	G2/M
5.4 ± 3.9	34.6 ± 2.6	18.0 ± 1.4	42.0 ± 3.4

Sub-G1	G1	S	G2/M
68.4 ± 3.9*	14.1 ± 0.6*	4.0 ± 0.2*	13.5 ± 2.2*

Supplementary Figure S2: CFZ increases the sub-G1 phase of the cell cycle in MM cell lines. U266 and RPMI cell lines were treated with CFZ for 24 h and cell cycle analysis was performed. Histograms are representative of one of three separate experiments. The values represent the percentage of cells in each phase and are expressed as mean ± SD. *$p<0.05$ vs vehicle treated cells.

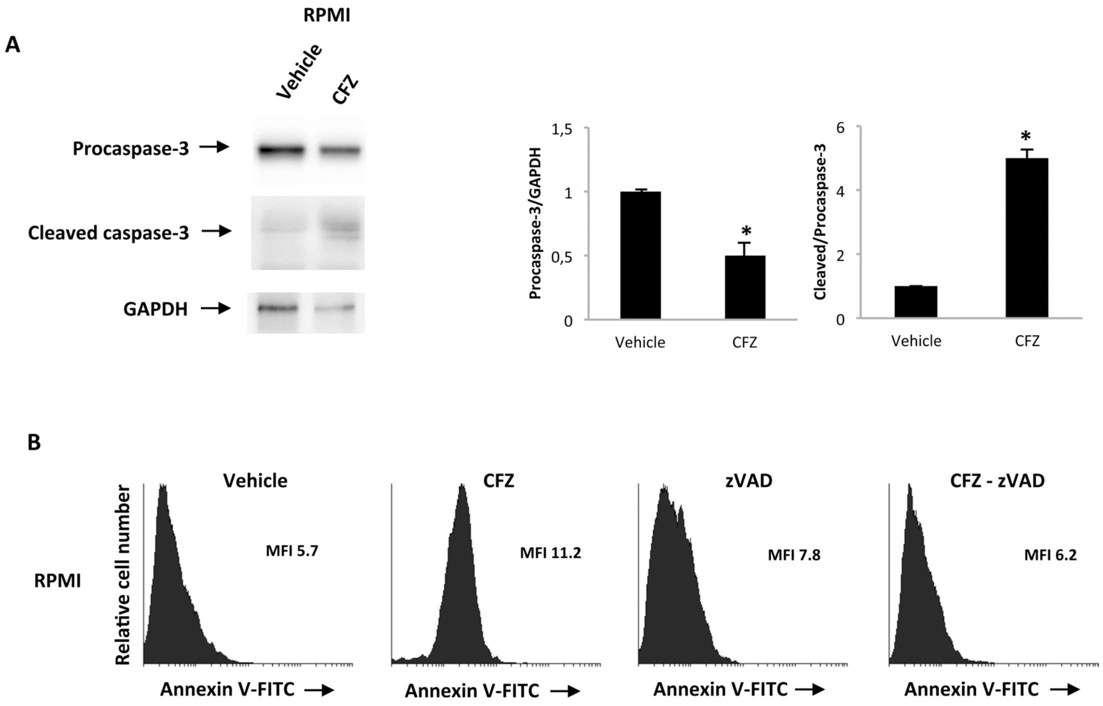

Supplementary Figure S3: CFZ induces apoptotic cell death in the RPMI cell line. A. Lysates from the RPMI cell line treated with CFZ 7.5 nM for 72 h were analyzed for caspase-3 protein level by western blot analysis. GAPDH protein levels were evaluated as the loading control. Blots are representative of three separate experiments. Bars represent the densitometric analysis. *p<0.01 vs vehicle cells. **B.** RPMI cell lines were pretreated with 5 mM zVAD for 1 h and then treated with 7.5 nM CFZ for 72 h. The percentage of Annexin V positive cells was determined by FACS analysis. Histograms are representative of one of three separate experiments. MFI, mean fluorescence intensity.

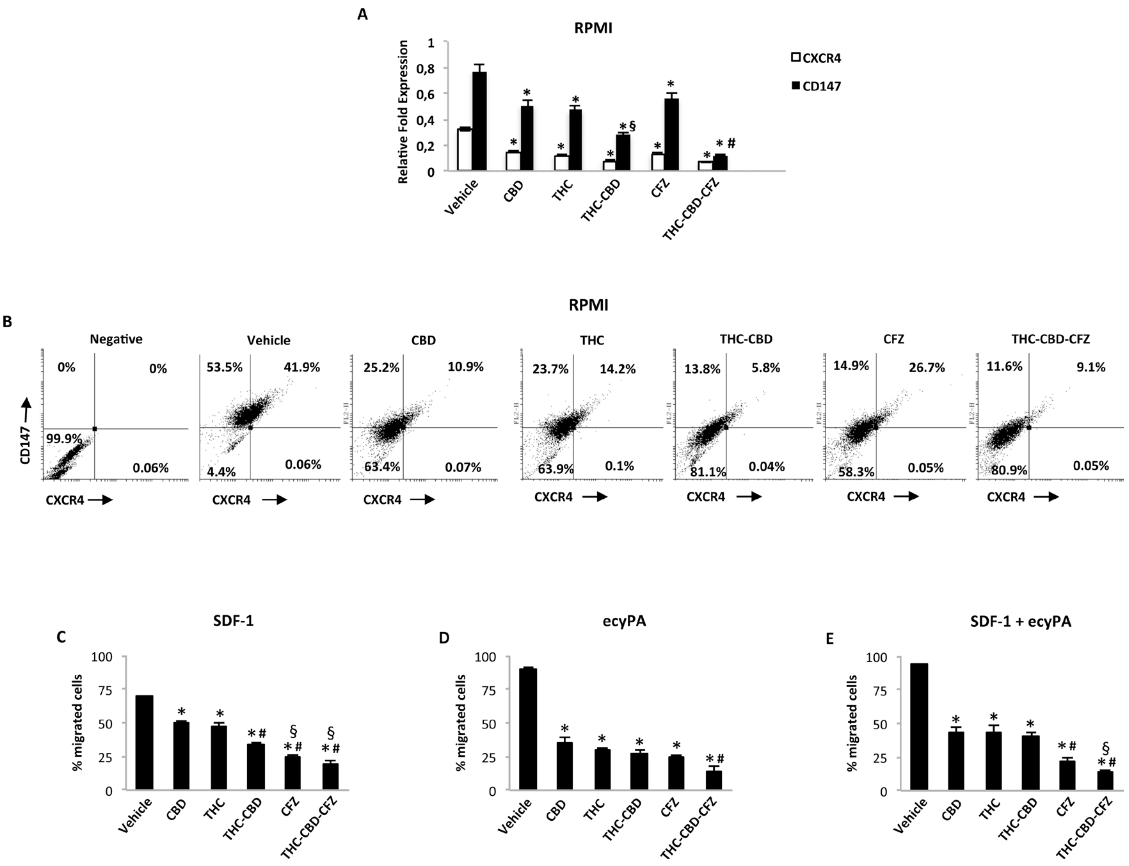

Supplementary Figure S4: THC, CBD and CFZ inhibit cell migration in the RPMI cell line. A. RPMI cells were treated with CBD 12.5 µM, THC 12.5 µM, CFZ 7.5 nM alone or in combination for 24 h. CXCR4 and CD147 mRNA levels were determined by qRT-PCR. GAPDH was used for normalization. Data are expressed as relative fold with respect to vehicle treated cells used as the control. Data are expressed as mean ± SD. *p<0.01 vs vehicle; §p<0.01 vs THC, CBD, CFZ alone; #p<0.05 vs THC, CBD, CBD-THC, CFZ. **B.** CXCR4 and CD147 expression was analyzed by fow cytometry on RPMI cell line treated as described above. Representative dot plots illustrate the double fluorescence. Numbers represent the percentage of cells in each quadrant. Data are representative of 1 of 4 independent experiments. **C-E.** Cell migration was analysed by transwell migration assays. Data represent the percentage of migrated RPMI cells and are expressed as mean ± SD. In C: *p<0.01 vs vehicle; #p<0.01 vs THC, CBD; §p<0.01 vs THC-CBD. In D: *p<0.01 vs vehicle; #p<0.01 vs THC, CBD, THC-CBD, CFZ. In E: *p<0.01 vs vehicle; #p<0.01 vs THC, CBD, THC-CBD; §p<0.01 vs CFZ.
